# Beyond troponins: Emerging diagnostic significance of novel markers in NSTEMI

**DOI:** 10.21542/gcsp.2024.33

**Published:** 2024-08-01

**Authors:** Amit Varshney, Vidya S. Ram, Pankaj Kumar

**Affiliations:** 1 Department of General Medicine, Noida International Institute of Medical Sciences, Gautam Budh Nagar, Uttar Pradesh, India; 2 Department of Medicine, Uttar Pradesh University of Medical Sciences, Saifai, Etawah, Uttar Pradesh, India

## Abstract

**Objective:** This study aims to comprehensively analyze a multiple-marker panel consisting of 55 morphofunctional and biochemical markers in 123 patients diagnosed with non-ST-segment elevation myocardial infarction (NSTEMI). The goal of this study was to identify novel pathogenetic landmarks and diagnostic predictors associated with NSTEMI.

**Methods:** The study includes 123 patients diagnosed with NSTEMI based on ESC Guidelines criteria. Clinical characteristics, morphofunctional markers, and serum levels of 53 biochemical markers related to inflammation, oxidative stress, endothelial dysfunction, cellular injury, hemostasis, and myocardial remodeling were assessed. A control group of 47 healthy individuals was included for comparison.

**Results:** NSTEMI patients exhibited an activated inflammatory status, oxidative stress, and endothelial dysfunction. Notable increases in inflammation markers, alterations in adipokines, and changes in oxidative stress markers were observed. Endothelial dysfunction markers indicated vascular remodeling and dysfunction. Cellular injury markers, including cMyBP-C, suggested myocardial necrotic injury. Hemostasis markers showed impaired anticoagulant systems, and ECM remodeling markers indicated increased matrix metalloproteinases.

**Conclusion:** The multiple-marker panel provides insights into novel pathogenetic entities associated with NSTEMI. Markers such as MPO, MMP-8, E-selectin, PhA2, Ang 2, FE, MF, and cMyBP-C demonstrate potential diagnostic and prognostic value. This comprehensive analysis enhances our understanding of NSTEMI pathogenesis and offers potential targets for therapeutic interventions.

## Introduction

Non-ST-elevation myocardial infarction (NSTEMI) poses a significant and complex challenge in cardiology for several compelling reasons. First, the prevalence of NSTEMI within the spectrum of acute coronary syndrome ranges widely, accounting for 35–65% of cases^1^. Despite this high occurrence, the intricate nature of NSTEMI remains a focal point owing to its multifaceted pathogenesis and clinical implications.

Another crucial aspect underscoring the gravity of NSTEMI is the mortality rate within the initial 12 months after revascularization, which equals or surpasses that of the more imminent ST-segment elevation myocardial infarction (STEMI). Recent studies^2^, emphasize the elevated risk of major adverse cardiovascular events (MACE) associated with NSTEMI, surpassing that of STEMI over a 3-year follow-up period.

The pathophysiological understanding of the evolution of NSTEMI introduces ambiguity on various fronts. One notable area of uncertainty involves the pathogenetic landmarks associated with the development of subendocardial infarction in cases of subtotal(75–90%) or total occlusion of the coronary arteries. This phenomenon, observed in a quarter of NSTEMI cases, raises questions about the interplay between atheroma progression in the “culprit” artery and the processes of vasculogenesis and angiogenesis^3^. These mechanisms significantly contribute to collateral development and influence the extent of myocardial injury.

Additionally, the pathogenetic landmarks related to impairment of coronary microcirculation remain elusive. Even in the presence of well-developed collaterals through vasculogenesis and/or angiogenesis, there are instances in which microcirculatory dysfunction leads to infarct development. The patterns of coronary microcirculation involvement, including endothelial alternative patterns with a vasospastic component or thromboembolic patterns, add further layers of complexity to the understanding of NSTEMI pathogenesis.

Furthermore, uncertainties persist regarding the post-infarction evolution of NSTEMI patients. This encompasses pathological remodeling of the myocardium, coronary vessels, and peripheral regions, elevating the risk of cardiac death and MACE during extended follow-up periods. In light of these complexities, ongoing research strives to unravel the intricate pathophysiological landscape of NSTEMI and pave the way for more effective management and improved outcomes in affected individuals.

The goal is not only to identify key predictors for accurate prognosis of post-infarction outcomes, but also to support therapeutic strategies that can reduce long-term mortality and major adverse cardiac events. Additionally, highlighting diagnostic value markers could facilitate the triage of NSTEMI patients when admission troponin levels are below the 99th percentile and TrT or TrI is to be determined repeatedly, according to the European guidelines for managing NSTEMI patients^4^.

The multiple marker panel is considered in this context as a feasible and efficient tool for the comprehensive research of the pathogenetic interface of NSTEMI, particularly if it involves biomarkers that, according to contemporary pathophysiological concepts, are related to leading pathological processes in circulatory homeostasis, such as inflammation, oxidative stress, endothelial dysfunction, hemostatic disruption, etc.

## Aim

As part of the multiple marker panel employed for patients with NSTEMI, the aim of this study was to assess the circulating levels of key cardiovascular pathology markers at admission. This was undertaken to delineate the pathogenetic linkage and identify diagnostic indicators.

## Material and Methods

The study was conducted on a sample of 123 patients diagnosed with NSTEMI based on the criteria outlined in the ESC Guidelines. Patients were included in the study based on the following criteria: (1) age ≥18 years; (2) confirmed diagnosis of NSTEMI, established according to the definition provided by the European Society of Cardiology (ESC) Guidelines and the universal definition of myocardial infarction. The general clinical characteristics of the patients are presented in Table 1.

**Table 1:** Basic clinical characteristics of the sampled patients.

**Demographic characteristics**	
Age, years (*M*±*m*)	67.8 ± 11.1
Women, % (*n*)	41.4% (51)
Men, % (*n*)	58.6% (72)
BMI, kg/m^2^	28.5 ± 4.6
**Risk Factors, % (*n*)**	
Hypertension	91.9% (113)
Hypercholesterolemia	71.2% (88)
Diabetes Mellitus	36.6% (45)
Smoker	26.8% (33)
− Current	16.3% (20)
- Previous	10.6% (13)
**Cardiovascular History, % (*n*)**	
Atrial Fibrillation	18.6% (23)
Heart Failure	50.4% (62)
Previous Myocardial Infarction	27.6% (34)
- with Q wave	16.3% (20)
- without Q wave	11.3% (14)
Confirmed Peripheral Arterial Disease	8.1% (10)
Stroke	10.6% (13)
Coronary Artery Bypass Grafting	3.2% (4)
**Non-Cardiac Comorbidities, % (*n*)**	
Active Cancer	0.8% (1)
Chronic Obstructive Pulmonary Disease	7.3% (9)
Chronic Renal Disease	14.6% (18)
**Pre-hospital Treatment, % (*n*)**	
Aspirin	47.9% (59)
Clopidogrel	7.1% (9)
Dual Anti-Platelet therapy	5.7% (7)
Oral Anticoagulants	5.7% (7)
ACE-I/ARB	52.8% (65)
Beta-blockers	39.0% (48)
Spironolactone	11.3% (14)
Statins	12.2% (15)
- Low Dose	5.7% (7)
- Standard Dose	5.7% (7)
- High Dose	0.8% (1)
Diuretics	19.5% (24)

**Notes.**

ACE-I/ARB Angiotensin Converting Enzyme Inhibitors/Angiotensin Receptor Blockers

Table 1 shows the demographic and clinical characteristics of the study population. The mean age of the patients was 67.8 years with a standard deviation of 11.1 years. The gender distribution is 41.4% women (51 individuals) and 58.6% men (72 individuals). The average BMI is 28.5 kg/m^2^ with a standard deviation of 4.6.

Regarding risk factors, 91.9% (113 individuals) had hypertension, 71.2% (88) had hypercholesterolemia, 36.6% (45) had diabetes mellitus, 26.8% (33) were smokers, 16.3% (20) were current smokers, and 10.6% (13) had previously smoked.

In terms of cardiovascular history, 18.6% (23) have atrial fibrillation, 50.4% (62) have heart failure, and 27.6% (34) have had a previous myocardial infarction, with 16.3% (20) having Q-wave infarctions and 11.3% (14) without Q-wave. Confirmed peripheral arterial disease is present in 8.1% (10), stroke in 10.6% (13), and 3.2% (4) have had coronary artery bypass grafting.

Non-cardiac comorbidities include active cancer in 0.8% (1), chronic obstructive pulmonary disease in 7.3% (9), and chronic renal disease in 14.6% (18).

Regarding pre-hospital treatment, 47.9% (59) were on aspirin, 7.1% (9) on clopidogrel, 5.7% (7) on dual anti-platelet therapy, 5.7% (7) on oral anticoagulants, 52.8% (65) on ACE inhibitors/ARBs, 39.0% (48) on beta-blockers, 11.3% (14) on spironolactone, 12.2% (15) on statins (with 5.7% on low dose, 5.7% on standard dose, and 0.8% on high dose), and 19.5% (24) on diuretics.

Morphofunctional markers, including the intima-media thickness of the common carotid artery and the flow-mediated dilation rate of the brachial artery, were determined using Doppler echocardiography. Biochemical research included the determination of serum levels of 53 markers related to 6 pathophysiological entities.

### Inflammation

1. IL-1, IL-6, IL-8, IL-4, IL-10, IL-33, interleukin 1 receptor-like 1 (ST-2), myeloperoxidase (MPO), leptin, adiponectin, omentin-1, resistin, TNF- *α*, hsCRP, E-selectin, and heregulin-1-beta.

### Oxidative stress

1. MDA (malondialdehyde), AOPP (advanced oxidation protein products), TAA (total antioxidant activity), SOD (superoxide dismutase), catalase, GPR (glutathione peroxidase), and GR (glutathione reductase).

### Endothelial dysfunction

1. PhA2 (phospholipase A2), Apolipoprotein A [Apo(a)], IMC of the carotid (intima-media thickness of the carotid artery), FMD (flow-mediated dilation rate of the brachial artery), FE (endothelial cell fragments), EPC (endothelial progenitor cells), NO (nitric oxide), S-nitrosothiols, Ang 2 (angiopoietin 2).

### Cellular injury

1. TrT (Troponin-T), FABP (fatty acid-binding protein in the heart), cystatin C, cMyBP-C (cardiac myosin-binding protein C).

### Hemostasis

1. MF (fibrin monomers), D-dimers, protein C and S, antithrombin III, AP (plasminogen activator), PAI-1 (plasminogen activator inhibitor-1).

### Myocardial and extracellular matrix (ECM) remodeling

1. Galectin-3, PINP (type I collagen synthesis marker), PIIINP (type III collagen synthesis marker), TIMP-1 (tissue inhibitors of metalloproteinases type-1), TIMP-3 (tissue inhibitors of metalloproteinases type-3), cardiotrophin, MMP-2, MMP-3, MMP-8, MMP-9, NT-Pro-BNP.

The control (or reference) group was comprised of 117 individuals who appeared to be in good health.

Statistical analysis of the obtained numerical data involved determination of the mean (M) and standard deviation (SD). When comparing indices between groups (control and NSTEMI patients), the discrepancy was considered significant when the *p*-value was less than 0.05.

## Results

The serum values of inflammatory markers at admission in patients with NSTEMI are presented in Table 2.

**Table 2:** Serum content of inflammation markers.

**Marker**	**Control group**	**NSTEMI group**	**Deviations**	***p*-value**
hsCRP, mg/L	1.2 ± 0.7	8.1 ± 1.5		<0.001
IL-1, pg/ml	5.88 ± 1.2	8.65 ± 1.4	+48%	<0.01
IL-4, pg/ml	4.12 ± 0.9	2.78 ± 0.6	−32%	<0.01
IL-6, pg/ml	4.65 ± 0.8	7.22 ± 1.1	+55%	<0.001
IL-8, pg/ml	4.33 ± 0.7	6.55 ± 1.1	+51%	<0.001
IL-10, pg/ml	7.19 ± 1.1	3.54 ± 0.8	−51%	<0.001
IL-33, pg/ml	3.76 ± 0.8	3.45 ± 0.6	−9%	>0.05
ST-2, pg/ml	2.62 ± 0.9	2.38 ± 0.5	−9%	>0.05
TNF- *α*, pg/ml	6.03 ± 0.9	9.72 ± 1.3	+61%	<0.001
MPO, U/ml	29.15 ± 4.47	74.8 ± 11.3	+156%	<0.001
E-Selectin, ng/ml	69.25 ± 4.15	105.63 ± 13.2	+53%	<0.001
Leptin, ng/ml	10.55 ± 2.1	12.83 ± 1.9	+21%	<0.05
Adiponectin, ng/ml	7.62 ± 1.5	5.87 ± 1.2	−23%	<0.05
Omentin-1, ng/ml	2.94 ± 0.8	5.34 ± 0.9	+81%	<0.001
Resistin, pg/ml	6.56 ± 1.1	9.18 ± 1.6	+40%	<0.01
Heregulin-1b, pg/ml	5.16 ± 0.9	3.85 ± 0.6	−24%	<0.01

**Notes.**

hsCRP high-sensitivity C-reactive protein IL Interleukin TNF-*α* Tumor Necrosis Factor alpha ST-2 interleukin 1 receptor-like 1 MPO myeloperoxidase

**Table 3:** Serum content of oxidative stress markers.

**Marker**	**Control group**	**NSTEMI group**	**Deviations**	***p*-value**
MDA, µmol/L	4.52 ± 0.8	7.29 ± 1.3	+61%	<0.001
AOPP, µmol/L	42.31 ± 8.5	71.35 ± 11.5	+67%	<0.001
TAA, mM/L	0.62 ± 0.6	0.39 ± 0.5	−37%	<0.001
SOD, u/c	1329 ± 209	884 ± 118	−33%	<0.01
Catalase, µmol/L	25.61 ± 3.9	19.44 ± 2.9	−25%	<0.01
GPR, nM/s.L	237.6 ± 36.6	185.2 ± 28.6	−22%	<0.01
GR, nM/s.L	115.4 ± 21.3	88.2 ± 18.8	−23%	<0.01

**Notes.**

MDA malondialdehyde AOPP advanced oxidation protein products TAA total antioxidant activity SOD superoxide dismutase GPR glutathione peroxidase GR glutathione reductase

**Table 4:** Serum content of endothelial dysfunction markers.

**Marker**	**Control group**	**NSTEMI group**	**Deviations**	***p*-value**
IMC, mm	0.95 ± 0.4	12.37 ± 1.9	+30%	<0.01
FMD, %	13.64 ± 2.3	9.78 ± 1.7	−28%	<0.01
PhA2, ng/ml	183.8 ± 28.7	344.5 ± 41.6	+88%	<0.001
Apo (a), µmol/L	44.61 ± 8.5	72.48 ± 11.5	+63%	<0.001
FE, cells/ml	8.8 ± 2.3	17.1 ± 4.2	+94%	<0.001
EPC, cells/µL	489.3 ± 59.8	318.4 ± 50.6	−35%	<0.01
Ang 2, pg/ml	2106 ± 33	3698 ± 40	+76%	<0.001
NO, µM/L	68.21 ± 10.5	49.46 ± 8.5	−28%	<0.05
S-nitrosothiols, µM/L	3.77 ± 0.7	2.65 ± 0.2	−30%	<0.01

**Notes.**

PhA2 phospholipase A2 Apo (a) Apolipoprotein A IMC of the carotid intima-media thickness of the carotid artery FMD flow-mediated dilation rate of the brachial artery) FE endothelial cell fragments EPC endothelial progenitor cells Ang 2 angiopoietin 2 NO nitric oxide

The obtained results suggest an activated inflammatory status, as evidenced by significant alterations in 14 of the 16 explored markers, except for IL-33 and ST-2. Notably, the average serum hsCRP level exceeded 8 mg/L, with an increase in major pro-inflammatory interleukins (IL-1, IL-6, and IL-8) ranging from to 48–55% and a 61% increase in TNF- *α*. The anti-inflammatory markers IL-4 and IL-10 showed declines of 32% and 51%, respectively, compared to the control values. Additionally, myeloperoxidase, a neutrophil marker, increased by 156% along with a 53% increase in E-selectin levels, a marker of polymorphonuclear transendothelial passage.

In the adipokine set, there was a significant decrease in anti-inflammatory markers adiponectin and heregulin-1b by 23–24%, whereas pro-inflammatory adipokines leptin, omentin-1, and resistin increased. Omentin-1 exhibited the most substantial rise at 81%, while leptin and resistin increased by 23% and 40%, respectively, compared to control levels.

The serum values of oxidative stress markers at admission in NSTEMI patients are presented in Table 3.

The results suggest an association between an augmented inflammatory response and oxidative stress activation, with markers of lipid and protein peroxidation (malondialdehyde and advanced oxidation protein products) elevated by 61–67%. This oxidative stress activation may be linked to decreased antioxidant defense, indicated by a 22–33% reduction in key antioxidant enzymes (SOD, catalase, GPR, and GR) and a 37% decrease in total antioxidant activity (TAA) compared to controls.

Inflammation and oxidative stress are important components that may contribute to endothelial dysfunction, which is a potential mechanism underlying myocardial ischemic injuries. These findings warrant further investigation to confirm these associations and better understand the interplay between inflammation, oxidative stress, and endothelial dysfunction in NSTEMI patients.

The initial levels of key markers indicating endothelial dysfunction in patients with NSTEMI are detailed in Table 4.

Evaluation of morphofunctional markers suggests notable endothelial vascular dysfunction and peripheral vascular remodeling in patients with NSTEMI. The intima-media thickness of the carotid artery was 30% greater, and the flow-dependent dilation rate of the brachial artery was 28% lower than that of the controls.

The correlation between biochemical and morphofunctional markers of endothelial dysfunction is evident. Markers of endothelial inflammatory lesions and atherogenicity, such as phospholipase A2 and circulating fragments of endotheliocytes, increased by 88–94%. Serum levels of Apo (a) and angiopoietin 2 also rose by 63–76%. A 35% reduction in endothelial progenitor cells may suggest compromised re-endothelialization. A moderate decrease in nitric oxide and endogenous S-nitrosothiols by 28–30% might indicate alterations in the endothelial control of coronary reactivity.

Estimating myocardial necrotic injury remains a cornerstone in diagnosing NSTEMI with troponins as reliable biomarkers. Other intracellular markers released into the blood during cardiomyocyte necrosis could serve as supplementary diagnostic references for troponins, particularly when early troponin levels do not exceed the 99th percentile. These findings are exploratory and suggest the need for further investigation into the complex interplay of these markers in NSTEMI.

The serum values of cellular injury markers at admission in NSTEMI patients are presented in Table 5.

**Table 5:** Serum content of cellular injury markers.

**Marker**	**Control group**	**NSTEMI group**	**Deviation**	***p*-value**
TrT, ng/L	0	389.4 ± 76.5		<0.001
H-FABP, ng/ml	2.64 ± 0.84	6.13 ± 1.3	+132%	<0.001
cystatin C, mg/L	0.68 ± 0.3	1.15 ± 0.6	+69%	<0.001
cMyBP-C, ng/L	91.44 ± 14	763.3 ± 128	8.38 times	<0.001

**Notes.**

TrT Troponin T H-FABP fatty acid-binding protein in the heart cMyBP-C cardiac myosin-binding protein C

The serum content of fatty acid-binding protein in the heart, an intracellular marker, was found to be increased by 132% in NSTEMI patients compared to controls. Cystatin C, another small intracellular marker, increased by 69%. Particularly noteworthy is the 8.38-fold increase in the circulating level of cardiac myosin-binding protein C (cMyBP-C). The physiopathological course of NSTEMI is associated with thromboembolic conditions of small-caliber coronary arteries and arterioles in the subendocardial myocardium. Therefore, assessing the functional feasibility of the hemostatic system may have conceptual and diagnostic implications.

The admission values of hemostasis markers in NSTEMI patients are presented in Table 6.

**Table 6:** Serum content of hemostasis markers.

**Marker**	**Control group**	**NSTEMI group**	**Deviation**	***p*-value**
FM, mg/ml	4.7 ± 0.8	9.5 ± 1.3	+102%	<0.001
D-dimer, mg/ml	0.29 ± 0.05	0.41 ± 0.08	+41%	<0.001
Protein C, %	85.4 ± 10	59.4 ± 7.6	−30%	<0.001
Protein S, %	88.1 ± 9.9	47.7 ± 6.4	−46%	<0.001
Antithrombin III, %	92.6 ± 11	73.4 ± 6.3	−22%	<0.001
PA, u/ml	11.4 ± 2.7	6.6 ± 1.8	−42%	<0.001
PAI-1, u/ml	2.8 ± 0.4	4.1 ± 0.7	+46%	<0.001

**Notes.**

FM fibrin monomers PA plasminogen activator PAI-1 plasminogen activator inhibitor-1

The analysis of hemostasis markers delineates the impairment of the anticoagulant system, as evidenced by a reduction in the serum levels of protein C, protein S, and antithrombin III by 22–46%. Consequently, the coagulant system intensifies and the 102% increase in fibrin monomers justifies this phenomenon. The elevation of D-dimer levels by 41% signifies the presence of a fibrin thrombus, although its cleavage with the formation of D-dimer occurs against the background of diminished fibrinolysis activity, as the tissue plasminogen activator (AP) level is reduced by 42%, and its inhibitor, on the contrary, increased by 46%.

The post-infarction evolution of NSTEMI and STEMI is indispensable for the quality of myocardial and extracellular matrix (ECM) remodeling. Highlighting specific patterns in the expression of key markers in this process can be a conceptual element and a valuable prognostic reference.

The admission values of myocardial remodeling and ECM markers in NSTEMI patients are presented in Table 7.

**Table 7:** Serum content of myocardial remodeling and ECM markers.

**Marker**	**Control group**	**NSTEMI group**	**Deviation**	***p*-value**
Galectin-3, ng/ml	4.9 ± 0.7	8.2 ± 1.1	+67%	<0.001
PINP, ng/ml	36.7 ± 5.6	25.2 ± 4.1	−31%	<0.001
PIIINP, µg/L	5.14 ± 0.6	3.91 ± 0.8	−25%	<0.001
TIMP-1, ng/ml	26.8 ± 4.2	41.9 ± 6.7	+56%	<0.001
TIMP-3, µg/L	8.8 ± 1.3	13.1 ± 2.2	+49%	<0.001
Cardiotrophin, pg/ml	64.7 ± 10.2	97.8 ± 12.8	+51%	<0.001
MMP-2, ng/ml	2.9 ± 0.4	4.1 ± 0.7	+41%	<0.001
MMP-3, ng/ml	1.7 ± 0.3	2.2 ± 0.6	+29%	<0.05
MMP-8, ng/ml	28.87 ± 4.65	54.20 ± 7.62	+88%	<0.001
MMP-9, ng/ml	14.3 ± 3.6	20.2 ± 4.8	+41%	<0.001
NT-Pro-BNP, pg/ml	147.8 ± 21.2	524.3 ± 79.8	+256%	<0.001
Copeptin, pmol/L	19.5 ± 4.15	30.4 ± 5.21	+56%	<0.001

**Notes.**

PINP (N- terminal propeptide of collagen type I) PIIINP (N- terminal propeptide of collagen type III) TIMP-1 (tissue inhibitors of metalloproteinases type-1) TIMP-3 (tissue inhibitors of metalloproteinases type-3) MMP Matrix Metalloproteinases NT-Pro-BNP N-terminal pro–B-type natriuretic peptide

In the spectrum of explored markers at the admission of patients with NSTEMI, an observed trend was the increased expression of matrix metalloproteinases (MMP) in the ECM, along with elevated markers for the degradation of fibrillar collagen type I (TIMP-1) and collagen type III (TIMP-3) by 49–56%. Notably, MMP-8 showed the highest increase at 88%, whereas the increments in MMP-2, MMP-3, and MMP-9 ranged between 29–41%. Concurrently, there was a minor decrease in the synthesis markers of collagen types I and III (PIMP and PIIIMP), up to 31%.

Myocardial remodeling appears to be associated with neuroendocrine activation in the myocardium and brain, which is potentially triggered by circulatory insufficiency. The serum content of NT-Pro-BNP is significantly elevated in patients with NSTEMI, and copeptin, a 39-amino acid peptide derived from the C-terminal end of arginine vasopressin pre-pro-hormone, exceeded the control level by 56%.

## Discussion

This study facilitated the comprehensive exploration of a multiple-marker panel comprising 55 morphofunctional (*n*= 2) and biochemical (*n*= 53) markers applied to patients with NSTEMI, aiming to highlight novel pathogenetic landmarks and markers with predictive value for NSTEMI diagnosis. In 123 patients with NSTEMI, the circulating levels of admission markers imminent to pathologic processes leading to circulatory dyshomeostasis, such as inflammation, oxidative stress, endothelial dysfunction, hemostasis impairment, myocardial remodeling, and extracellular matrix remodeling, were determined.

Beyond providing an overview of the role of these pathologic processes in the evolution of NSTEMI, the true conceptual value of this study, consolidated within the multiple marker panel analysis, lies in justifying the diagnostic relevance of markers reflecting distinct pathogenetic entities. For instance, in the spectrum of intelligible changes in inflammatory markers, elevation of myeloperoxidase (MPO) by 156% is of interest not only because of the degree of increase but also because this pro- inflammatory marker derived from neutrophils infiltrates the myocardial necrotic zone from the onset, conclusively ensuring necrosis expansion through NETosis^5,6^. Therefore, MPO, which is specific to neutrophils, serves as a predictor of myocardial necrosis, making it a potential therapeutic target.

The alternative involvement of polymorphonuclear cells in the myocardium is further evidenced by the release of MMP-8 from neutrophils, and in our study, this marker showed the most pronounced elevation (+88%) compared to control, in contrast to other metalloproteinases such as MMP-2, MMP-3, and MMP-9. Thus, the estimation of MPO and MMP-8 elucidates not only the pathogenetic importance of NETosis but also provides notable contributions to the diagnosis of NSTEMI and the risk of necrotic zone expansion in the post-infarction evolution of the patient^6–9^.

The transendothelial passage of neutrophils in the necrotic zone of the myocardium is facilitated by selectins (E, L, P) and integrins. The serum content of E-selectin increased by 53%, consistent with the changes observed in MPO and MMP-8. Remarkably, the results of the SELECT-ACS trial^10^, which evaluated the effect of inclacumab, a specific antagonist of P-selectin (a leukocyte adhesion molecule), on the evolution of NSTEMI, established a reduction in troponin levels, which is a consequence of reducing the necrotic zone. Therefore, MPO as a specific marker of NETosis can be separated from the set of inflammation markers as a predictor of NSTEMI diagnosis and prognosis and is an important therapeutic target. M. Ali et al. ^11^ demonstrated in an experimental study that inhibiting MPO reduced the myocardial necrotic zone and improved the functional and structural remodeling of the heart.

The exposition of explored oxidative stress markers does not allow for the segregation of a marker, similar to MPO, that excels in a pathogenetic entity closely associated with NSTEMI and gains support as a diagnostic and/or prognostic predictor.

In the spectrum of morphofunctional and biochemical markers appreciated for endothelial injury and dysfunction, PhA2, Ang 2, and FE represent coherent pathogenetic entities, reflecting (1) the inflammatory activity of the coronary artery intima supported by the imminent macrophage of phospholipase A2 leading to endothelial dysfunction^12^; (2) negative remodeling of the coronary vessels, including Ang 2 at the microcirculation level^13^; and (3) endotheliocyte injury resulting in the circulation of FE (microparticles or endothelial debris) with a strong prothrombotic effect^14^. It is plausible to admit that, in assuming a diagnostic value for NSTEMI, these three markers must be estimated together since they mark a common pathological process and, therefore, a solitary mechanism, and the predictive power would be based on their elevation in the range estimated by us, 76–94%.

Moreover, the modification of these three markers in the context of activating prothrombotic processes against the background of endothelial dysfunction is closely related to the detected hemostasis impairment. Fibrin monomers (MF), which stood out with a more than double increase, can be a predictor of the prothrombotic pattern of coronary microcirculation dysregulation. MF is estimated as a predictor of the risk of long-term major adverse cardiovascular events (MACE) in patients with heart failure^15^. Unlike D-dimers, whose elevation depends on the activity of the fibrinolysis system, MF indicates an imbalance in the coagulant/anticoagulant system and is thus a more reliable marker of hemostasis impairment.

In conclusion, the current diagnosis of NSTEMI is based on troponin levels as a marker of necrotic myocardial cell injury. Additionally, patients with NSTEMI in our study had a serum level of cMyBP-C 8.38 times higher compared to the control marker. This protein, which binds to cardiac myosin, is a reliable marker of cardiomyocyte integrity and is released into the blood even with minimal myocardial injury^16–19^. Therefore, cMyBP-C can be regarded as a predictive marker for NSTEMI, especially in cases of faulty triage when troponin levels at admission do not exceed the 99th percentile. It is important to mention that S. Panotopoulos et al.^20^ consider cMyBP-C as a “Candidate Biomarker” of acute myocardial infarction, useful in differentiating NSTEMI or STEMI from unstable angina.

The elevated levels of H-FABP and cystatin C detected in NSTEMI patients are understandable, and the ESC Guidelines for managing patients with NSTEMI recommend the complementary use of these markers in biochemical diagnosis. The cMyBP-C marker can also be used as a reliable diagnostic marker.

### Potential clinical utility and validation of biomarker candidates

The potential clinical utility of the highlighted biomarker candidates lies in their ability to enhance the accuracy and timeliness of cardiovascular disease diagnosis. To validate these biomarkers as diagnostic tests, several steps are necessary. First, conducting studies with larger and more diverse cohorts is essential to confirm the findings and to ensure reliability across different populations. Longitudinal studies are needed to establish the temporal relationship between biomarker levels and disease progression to provide insights into their predictive value. Validation studies in independent cohorts are crucial for replicating findings and ensuring that biomarkers are not population-specific. Standardization of assays is critical for reproducibility and sensitivity, requiring optimized protocols for sample collection, processing, and analysis. Comparing new biomarkers with existing diagnostic tools will help to assess their added value in terms of sensitivity, specificity, and predictive values. Integration into clinical practice requires clear benefits, such as improved patient outcomes and cost-effectiveness, often necessitating clinical trials. Following these steps will rigorously validate biomarkers and potentially integrate them into clinical practice, enhancing diagnostic capabilities and patient care in cardiovascular medicine.

## Limitations

This study has several limitations that must be acknowledged. One primary limitation is the relatively small sample size of 123 patients with NSTEMI and 117 healthy controls, which may reduce the study’s statistical power and potentially lead to Type II errors. The limited sample size also raises concerns regarding the generalizability of the findings, as the study population predominantly included individuals from a single geographic region, which may not reflect broader and more diverse populations. Additionally, despite efforts to control for potential confounders, residual confounding factors, such as comorbid conditions, medication use, lifestyle factors, and genetic predispositions, could still influence the biomarker levels studied. The cross-sectional design of the study further limits the ability to establish causation between identified biomarkers and NSTEMI. Longitudinal studies are needed to track changes in biomarker levels over time and their correlation with clinical outcomes to provide stronger evidence of their prognostic value.

## Conclusion

The application and analysis of multiple marker panels in patients with NSTEMI have highlighted markers that signify novel pathogenetic entities with potential diagnostic value. These include: MPO as a marker of NETosis and the risk of necrotic zone expansion; PhA2, Ang 2, FE, and MF as markers of endothelial injury and dysfunction, as well as the prothrombotic pattern of coronary microcirculation dysregulation; and cMyBP-C as a marker of myocardial necrotic injury.

## Author Contributions

**Conceptualization:** Amit Varshney, and Pankaj Kumar. **Data curation:** Amit Varshney, Vidya S. Ram and Pankaj Kumar. **Formal analysis:** Amit Varshney, Vidya S. Ram, and Pankaj Kumar. **Funding acquisition:** Amit Varshney and Pankaj Kumar. **Investigation:** Amit Varshney, Vidya S. Ram, and Pankaj Kumar. **Methodology:** Amit Varshney and Vidya S. Ram. **Project administration:** Amit Varshney, Vidya S. Ram, and Pankaj Kumar. **Resources:** Amit Varshney and Pankaj Kumar. **Software:** Amit Varshney, Vidya S. Ram, and Pankaj Kumar. **Supervision:** Amit Varshney, Vidya S. Ram, and Pankaj Kumar. **Validation:** Amit Varshney. **Visualization:** Amit Varshney and Vidya S. Ram. **Writing - original draft:** Amit Varshney, Vidya S. Ram, and Pankaj Kumar. **Writing - review & editing:** Amit Varshney.
